# Intravascular CNS lymphoma: Successful therapy using high-dose methotrexate-based polychemotherapy

**DOI:** 10.1186/2162-3619-1-37

**Published:** 2012-12-05

**Authors:** Sied Kebir, Klaus Kuchelmeister, Pitt Niehusmann, Michael Nelles, Young Kim, Sharmilan Thanendrarajan, Niklas Schäfer, Moritz Stuplich, Frederic Mack, Björn Scheffler, Horst Urbach, Martin Glas, Ulrich Herrlinger

**Affiliations:** 1 Division of Clinical Neurooncology, Department of Neurology, University of Bonn Medical Center, Bonn, Sigmund-Freud-Straße 25, 53127 Bonn, Germany; 2 Department of Radiology, University of Bonn Medical Center, Bonn, Sigmund-Freud-Straße 25, 53127 Bonn, Germany; 3 Department of Neuropathology, University of Bonn Medical Center, Bonn, Sigmund-Freud-Straße 25, 53127 Bonn, Germany; 4 Department of Oncology, University of Bonn Medical Center, Bonn, Sigmund-Freud-Straße 25, 53127 Bonn, Germany; 5 Stem Cell Pathologies, Institute of Reconstructive Neurobiology, University of Bonn Medical Center, Bonn, Sigmund-Freud-Straße 25, 53127 Bonn, Germany

**Keywords:** Intravascular lymphoma, Intravascular CNS lymphoma, High-dose methotrexate-based polychemotherapy, Angioendotheliomatosis, Angiotropic lymphoma

## Abstract

Intravascular diffuse large B-cell lymphoma limited to the CNS (cIVL) is a very rare malignant disorder characterized by a selective accumulation of neoplastic lymphocytes (usually B cells) within the lumen of CNS blood vessels but not in the brain parenchyma. In the past, treatment of cIVL with anthracycline-based regimens was unsatisfactory with very short survival times. In the case of cIVL presented here, high-dose methotrexate-based polychemotherapy according to the Bonn protocol plus rituximab therapy was successful and led to a complete clinical and MRI remission which is ongoing 29 months after diagnosis.

## Background

Intravascular lymphoma, also known as intravascular lymphomatosis or angiotropic lymphoma and formerly known as malignant angioendotheliomatosis is a rare neoplastic disorder in which tumour cells are initially confined to the vascular lumen without parenchymal infiltration. While cases of systemic intravascular lymphoma are more frequently encountered, cases of intravascular lymphoma with restricted central nervous system (CNS) involvement (cIVL) are uncommon and only few patients that had been successfully treated have been reported so far [1-4]. We here present a case with a histologically confirmed cIVL that could be successfully treated with a high-dose methotrexate (HD-MTX) and rituximab-based chemotherapy regimen.

## Case presentation

**Figure 1:**
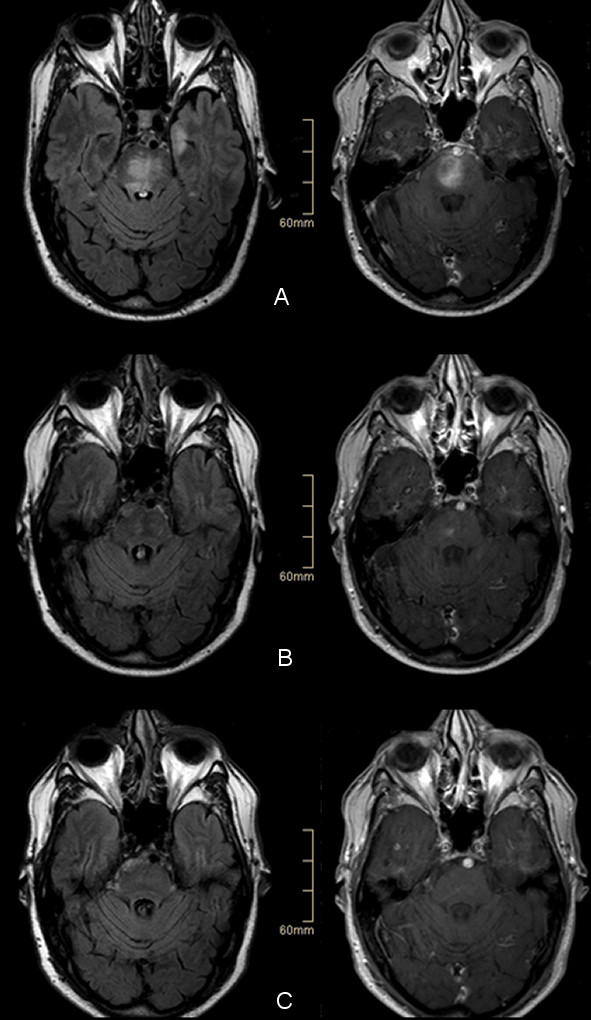
**MR imaging prior to and after HD-MTX-based chemotherapy (left column FLAIR, right column: contrast enhanced T1-weighted imaging)MR imaging prior to therapy (A) and at follow-up imaging at the end of 6 courses of chemotherapy with a strong reduction of contrast-enhancing lesions (B).** Nineteen months after initiation of treatment MR imaging showed complete regression of marked FLAIR hyperintensities and contrast enhancement in the brain stem **(C).**

**Figure 2:**
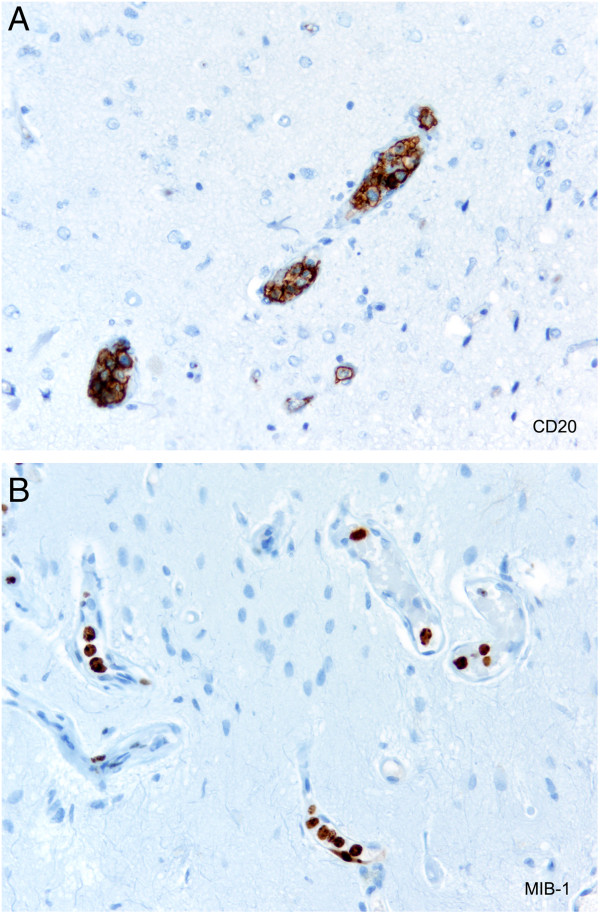
**Histological examination of the tissue obtained by stereotactic biopsy of the brain stem.** Histology revealed CD20-immunopositive intravascular lymphoma cells **(A)** with a very high proliferative activity in MIB-1 immunohistochemistry **(B).**

A 69-year-old male Caucasian patient presented with recurrent transient amnestic aphasia and gait ataxia. Physical examination at the time of referral did not reveal any further pathological findings. B symptoms were absent. Serum LDH levels were twice the upper limit of normal, all other serum chemistry and differential blood count was negative. Cerebrospinal fluid (CSF) analysis revealed a normal cell count, protein levels were within the reference range, no atypical cells were detected. Initial magnetic resonance imaging (MRI) revealed a contrast-enhancing lesion in the pons (Figure 1A) and additional involvement of the left temporomesial area. A stereotactic biopsy was performed and histology revealed a CD20-antigen-expressing intravascular lymphoma with high proliferative activity (Figure 2A, B). Immunohistological evaluation of B-cell differentiation markers showed a BCL-6+ and MUM-1+−status. Subsequent staging (i.e. examination of the chest, abdomen and pelvis by contrast-enhanced computed tomography (CT) scan, bone marrow biopsy, slit lamp examination of the eye, spinal tap) did not reveal any systemic or additional CNS involvement.

Chemotherapy according to the Bonn protocol was initiated in combination with rituximab therapy. The Bonn protocol comprises six 3-week courses with different combinations of HD-MTX (3 gm/m^2^ over 24 hours), ifosfamide, procarbazin, cytarabine, vinca alkaloids, and dexamethasone (for details see [5]). Rituximab was given at each course one day prior to the start of the HD-MTX infusion. During the 5th course, a transient and moderate increase in serum creatinine occurred, without a need for dose reduction in subsequent treatment courses. Vincristine was removed from the treatment protocol after development of mild signs of polyneuropathy. After the second course, the contrast-enhancing lesion showed already a partial remission; after the sixth course, only one small contrast-enhancing lesion remained that had to be qualified as unconfirmed complete remission since it further diminished in subsequent control MRIs without additional therapy (Figure 1A-C). The patient is now in complete clinical and radiographic remission 29 months after initial diagnosis of cIVL.

In this case report we demonstrate the successful therapy of a patient with cIVL, i.e. intravascular lymphoma limited to the CNS. The few reports available on the treatment of this medical condition are summarized in Table 1. All cIVL cases in which progression and death due to systemic failure was explicitly mentioned were not included here. In some cases, lymphoma-directed specific therapy was not applied or the treatment modality was not reported. In these cases, survival did not exceed 4 months [6-9]. Conventional chemotherapy with anthracyline-based protocols (i.e. CHOP in 3 patients), radiotherapy, or corticosteroid therapy was not successful [10-12]. Using anthracycline-based chemotherapy which is effective in systemic intravascular lymphoma does not penetrate the intact blood–brain barrier (BBB), overall survival rarely exceeded 6 months. Our case, on the other hand, is in line with reports demonstrating that BBB-penetrating HD-MTX-based regimens may have considerable efficacy. Seven patients treated with HD-MTX alone or in combination with CHOP survived 6–20 months [1,13,14]. In a separate study, three patients with cIVL receiving HD-MTX-based chemotherapy showed progression-free survival times of 2, 20 and 48 month [1-3]. One additional case report presented a patient receiving HD-MTX + R-CHOP followed by consolidation therapy with high-dose chemotherapy (thiotepa, busulfan, and cyclophosphamide) and autologous stem-cell rescue. This patient survived for at least 19 months after treatment [4]. It remains unclear why HD-MTX-based, i.e. blood–brain barrier (BBB)-penetrating therapy is needed for successful therapy of cIVL and which are the optimal combination partners for MD-MTX therapy. Also, it is unclear why regimens that do not penetrate the BBB but are effective in other forms of intravascular lymphoma are not successful in cIVL. This is particularly puzzling since all cIVL tumour cells are by histological definition located within the vessels and not beyond in the brain parenchyma.

**Table 1:** Summary of all patients with intravascular lymphomatosis limited to the CNS (cIVL) reported in the literature

**Author**	**Site of involvement**	**Neurological symptoms**	**Treatment**	**Outcome**
Baehring et al. [1]	Brain	Right hemiparesis, dysarthria	HD-MTX (induction 5, consolidation 10, maintenance 2)	CR 20 months after diagnosis
Baehring et al. [1]	Brain, spinal cord	Proximal spastic paraparesis, psychosis	HD-MTX (induction 12)	PR 18 months after diagnosis
Baehring et al. [1]	Brain, nerve roots	Dysarthria, gait disturbance, allodynia	HD-MTX initially (induction 6, consolidation 4), HD-MTX salvage (6 induction, 7 consolidation)	PR until 8 months after diagnosis: PR until 12 months after recurrence
Baehring et al. [1]	Brain	Cognitive decline, homonymous hemianopsia, ataxia	HD-MTX (induction 1 cycle)	Died of disease progression after first cycle of chemotherapy
Calamia et al. [15]	CNS	NA	m-BACOD	OS 16 months
Calamia et al. [15]	CNS	NA	Pro-MACE-CytaBOM	OS 44 months
Bergmann et al. [6]	Brain	Left-sided hemiparesis	NA	OS 2 months
Bergmann et al. [6]	Brain	Spastic paraparesis, left arm paresis	NA	OS 2 months
DiGiuseppe et al. [3]	Brain	Mental status changes	Pro-MACE-CytaBOM, ifosfamide/VP-16/cisplatin & whole brain irradiation (45 Gy)	CR 48 months after diagnosis
Kanda et al. [16]	CNS	Aphasia, apraxia	CHOP, VEMP, radiotherapy	OS one month
Aznar et al. [7]	CNS	Distal paresthesia of the lower limbs, paraparesis	NA	OS few months
Passarin [8]	Brain	Progressive cognitive deterioration, tetraparesis	NA	OS 3–4 weeks
Natali-Sora et al. [17]	CNS	Generalized tonic-clonic seizures	Cyclophosphamide, mitoxantrone, BCNU, methylprednisolone	CR 46 months after diagnosis
Liow et al. [18]	CNS	NA	CHOP	OS 13 months
Albrecht et al. [9]	Brain	Cognitive deterioration, aphasia	NA	OS few weeks
Ferreri et al. [10]	CNS	NA	CHOP (3 patients), CVP (one patient)	OS less than 4 months
Holmøy et al. [12]	Brain	vertigo, diplopia, left-sided hearing loss, aphasia	high-dose corticosteroid pulse therapy	OS 18 weeks
Momota et al. [14]	Brain	Left-sided hemiparesis	HD-MTX, whole brain irradiation	OS 6 months
**This case**	Brain	Transient amnestic aphasia, gait ataxia	Bonn protocol + rituximab	CR 29 months after diagnosis

Abbreviations: HD-MTX: high dose methotrexate; CR: complete remssion; PR: partial response; NA: not available; OS: overall survival; m-BACOD: cyclophosphamide, doxorubicin, vincristine, bleomycin, dexamethasone, methotrexate; Pro-MACE-CytaBOM: prednisone, methotrexate (with leucovorin rescue), doxorubicin, cyclophosphamide, etoposide, cytarabine, bleomycin, vincristine; VP-16: etoposide; CHOP: cyclophosphamide, doxorubicin, vincristine, prednisolone; VEMP: vincristine, cyclophosphamide, mercaptopurine, prednisolone; CVP: cyclophosphamide, vincristine and prednisone; Bonn protocol: HD-MTX, ifosfamide, procarbazin, cytarabine, vinca alkaloids, dexamethasone.

## Conclusion

Overall, on the base of our case and upon reviewing the literature, we recommend the use of HD-MTX-based polychemotherapy similar to HD-MTX-based protocols for primary (parenchymal) CNS lymphoma in patients with cIVL.

### Consent

Written informed consent was obtained from the patient for publication of this Case report and any accompanying images. A copy of the written consent is available for review by the Editor-in-Chief of this journal.

## Competing interests

The authors declare that they have no competing interests.

## Authors’ contributions

Conceived and designed therapy: UH MG. Performed neuroradiologic analysis: HU MN. Neuropathological diagnosis: KK PN. Wrote the paper: SK UH. Performed treatment and participated in collecting data: SK YK ST MS FM NS. All authors read and approved the final manuscript.
